# Neural representation in the auditory midbrain of the envelope of vocalizations based on a peripheral ear model

**DOI:** 10.3389/fncir.2013.00166

**Published:** 2013-10-21

**Authors:** Thilo Rode, Tanja Hartmann, Peter Hubka, Verena Scheper, Minoo Lenarz, Thomas Lenarz, Andrej Kral, Hubert H. Lim

**Affiliations:** ^1^Department of Otorhinolaryngology, Hannover Medical UniversityHannover, Germany; ^2^Department of Otorhinolaryngology, Berlin Medical University – ChariteBerlin, Germany; ^3^Department of Biomedical Engineering, University of MinnesotaMinneapolis, MN, USA

**Keywords:** auditory brainstem implant, cochlear implant, envelope, inferior colliculus, model, phase locking, speech, temporal code

## Abstract

The auditory midbrain implant (AMI) consists of a single shank array (20 sites) for stimulation along the tonotopic axis of the central nucleus of the inferior colliculus (ICC) and has been safely implanted in deaf patients who cannot benefit from a cochlear implant (CI). The AMI improves lip-reading abilities and environmental awareness in the implanted patients. However, the AMI cannot achieve the high levels of speech perception possible with the CI. It appears the AMI can transmit sufficient spectral cues but with limited temporal cues required for speech understanding. Currently, the AMI uses a CI-based strategy, which was originally designed to stimulate each frequency region along the cochlea with amplitude-modulated pulse trains matching the envelope of the bandpass-filtered sound components. However, it is unclear if this type of stimulation with only a single site within each frequency lamina of the ICC can elicit sufficient temporal cues for speech perception. At least speech understanding in quiet is still possible with envelope cues as low as 50 Hz. Therefore, we investigated how ICC neurons follow the bandpass-filtered envelope structure of natural stimuli in ketamine-anesthetized guinea pigs. We identified a subset of ICC neurons that could closely follow the envelope structure (up to ß100 Hz) of a diverse set of species-specific calls, which was revealed by using a peripheral ear model to estimate the true bandpass-filtered envelopes observed by the brain. Although previous studies have suggested a complex neural transformation from the auditory nerve to the ICC, our data suggest that the brain maintains a robust temporal code in a subset of ICC neurons matching the envelope structure of natural stimuli. Clinically, these findings suggest that a CI-based strategy may still be effective for the AMI if the appropriate neurons are entrained to the envelope of the acoustic stimulus and can transmit sufficient temporal cues to higher centers.

## INTRODUCTION

An auditory midbrain implant (AMI) designed for stimulation across the central nucleus of the inferior colliculus (ICC) was implanted in deaf patients who could not sufficiently benefit from a cochlear implant (CI; Lim et al., 2007, 2009). These patients have neurofibromatosis type 2, which is a genetic disease that leads to bilateral acoustic neuromas. Removal of the tumors usually leads to complete damage of the auditory nerves, and thus it is not possible to transmit acoustic information to the brain using a CI, which is designed to stimulate the auditory nerve. The AMI has been implanted in five patients. Encouragingly, the AMI has shown to be safe for over 6 years and has improved environmental awareness and lip-reading capabilities for the implanted patients. However, open-set speech understanding remains significantly lower for the AMI compared to the CI. Based on previous studies in animals and humans (Lenarz et al., 2006; Lim et al., 2009, 2013; Calixto et al., 2012; McKay et al., 2013), it appears that the AMI can transmit sufficient spectral cues when the electrode array is aligned along the tonotopic gradient of the ICC; however, the AMI cannot transmit sufficient temporal cues shown to be important for speech understanding (Shannon et al., 1995, 2004). The AMI currently uses a stimulation strategy designed for CIs in which the sound is processed through bandpass filters and the envelope of each filtered signal is used to amplitude-modulate an electrical pulse train presented to a frequency-aligned site. The question arises as to whether such a CI-based strategy is appropriate for the AMI.

For the CI, it is possible to position electrode sites in different locations along the cochlea to transmit sufficient spectral cues while presenting varying temporal patterns on each site to sufficiently transmit temporal cues for speech understanding (Shannon et al., 1995, 2004). In contrast, the ICC is a three-dimensional structure consisting of two-dimensional isofrequency laminae (Geniec and Morest, 1971; Malmierca et al., 1995; Oliver, 2005). Although sites can be positioned in each lamina to convey spectral cues, it is unclear if an amplitude-modulated pulse pattern delivered to only one site in each lamina would transmit sufficient temporal cues. Previous animal studies have shown complex and varying coding properties, including differences in temporal following abilities, for neurons along an ICC lamina (Semple and Aitkin, 1979; Stiebler, 1986; Ehret, 1997; Langner et al., 2002; Hage and Ehret, 2003; Holmstrom et al., 2010). Neurons sensitive to a similar frequency (i.e., with a similar best frequency, BF) can have dramatically different response patterns for the same stimulus. Furthermore, studies have shown that neurons within the ICC are no longer able to follow the fast fluctuations of a sound stimulus, particularly those above a few hundred hertz (Rees and Moller, 1987; Langner and Schreiner, 1988; Krishna and Semple, 2000; Frisina, 2001). Auditory nerve fibers can follow stimuli up to several thousand hertz (Johnson, 1980; Joris et al., 2004). Therefore, it has been proposed that temporal features have been converted, at least for faster components, into a rate code and/or population spiking pattern across ICC neurons, reflecting the complex transformations that have occurred through several nuclei from the auditory nerve (Langner and Schreiner, 1988; Langner et al., 2002; Joris et al., 2004; Wang et al., 2008; Huetz et al., 2011). These findings suggest that a CI-based stimulation strategy would not be appropriate for the ICC. Rather, electrode sites may need to be positioned fully across each lamina of the ICC and activated in various spatial and temporal patterns to transmit sufficient temporal cues. Implanting multiple shanks across the ICC to span each lamina increases surgical risk and identifying optimal ways to activate the numerous sites along each lamina would be challenging.

On the other hand, acoustic and CI studies in humans have shown that limited temporal cues, even down to 50 Hz, are sufficient for speech understanding in quiet (Shannon et al., 1995; Nie et al., 2006; Xu and Pfingst, 2008), which would be the initial goal for the AMI. ICC neurons are still capable of following the temporal pattern of sound stimuli above 50 Hz and even up to a few hundred hertz, at least for artificial click trains and amplitude modulated stimuli (Rees and Moller, 1987; Krishna and Semple, 2000; Frisina, 2001; Langner et al., 2002; Joris et al., 2004; Zheng and Escabi, 2008). Therefore, a CI-based strategy may be effective for the AMI, assuming that activation of just a subset of ICC neurons with a single site within each lamina is sufficient for restoring speech understanding. Although the AMI uses a CI-based strategy in the current patients, it is not clear if the limited performance is related to the stimulation strategy or if the AMI sites are just not activating the appropriate neurons in the ICC that would be able to transmit the necessary temporal cues to higher centers.

Previous animal studies investigating the envelope following capabilities of ICC neurons to natural stimuli generally investigated the overall response patterns averaged across neurons from different frequency and isofrequency locations (Suta et al., 2003; Woolley et al., 2006). These studies suggested that ICC neurons, as a population response, could approximately follow the envelope pattern of different vocalizations. One of these studies by Suta et al. (2003) also presented spiking responses for a few individual ICC neurons. However, further studies are needed to directly assess how well ICC neurons within different frequency regions follow the envelope structure of the vocalizations, and thus if it is possible to use a CI-based strategy for ICC stimulation. One challenge in achieving this goal is how to directly compare the ICC spiking response with the envelope structure of the stimulus. Sound is modified through the outer and middle ear and converted into basilar membrane motion at different frequency locations along the cochlea, undergoing several non-linear transformations (Rhode, 1971; Meddis et al., 2001; Sumner et al., 2003; Irino and Patterson, 2006; Jepsen et al., 2008). Thus, it would seem appropriate to directly compare the ICC responses to the envelope of this basilar membrane motion corresponding to the correct BF locations and incorporating the complex transformations through the peripheral system (e.g., frequency tuning characteristics along the basilar membrane, non-linear amplitude compression of the cochlea, and middle ear filtering). In addition to these peripheral effects, it is also possible to incorporate the transformations through the hair cell synapses and auditory nerve fibers to estimate the neural pattern that is observed at the input of the brain (Zhang et al., 2001; Bruce et al., 2003; Sumner et al., 2003; Zilany et al., 2009).

Based on the considerations presented above, we performed a detailed assessment in ketamine-anesthetized guinea pigs of how well ICC neurons across different frequency regions can follow the envelope structure of a diverse range of spectral and temporal features as found in species-specific vocalizations. We recorded neural activity across a large number of sites within the ICC by using multi-site electrode arrays to more effectively sample the different types of possible responses. We also incorporated a peripheral ear model specifically designed for the guinea pig that provides the basilar membrane signal for each frequency channel (i.e., velocity at a specific basilar membrane location; Meddis et al., 2001; Sumner et al., 2003). The envelope of this signal can then be compared to the responses recorded in the corresponding frequency region of the ICC. The effects associated with the hair cell synapses and auditory nerve fibers were not incorporated into our peripheral modeling because we were initially interested in treating the transformation from the basilar membrane to the ICC as a single black box without incorporating any “neural” components. In this way, we could directly assess if ICC neurons can accurately follow the BF-matched envelope of the stimulus that is actually driving the neural impulses into the brain. Later, we will incorporate additional components to more accurately reveal how information is coded from the basilar membrane to the ICC, expanding on models from previous studies (Hewitt and Meddis, 1994; Nelson and Carney, 2004; Guerin et al., 2006; Jepsen et al., 2008; Dugue et al., 2010). By incorporating a simple model of the peripheral ear, we were able to observe a surprisingly close resemblance between the BF-matched envelope of the stimulus and the spiking response of ICC neurons. There were many neurons that did not exhibit responses resembling the temporal structure of the stimulus. However, the existence of at least a subset of neurons spanning different frequency regions across the ICC that could closely follow the envelope of vocalizations suggests that CI-based stimulation strategies may still be successful for the AMI.

## MATERIALS AND METHODS

Basic surgical procedures and methods for neural recording and acoustic stimulation were similar to those presented in previous work (Lim and Anderson, 2006; Calixto et al., 2012) and performed in ketamine-anesthetized guinea pigs. For this study, we acoustically presented species-specific vocalizations to the animals and recorded the corresponding spike activity along the tonotopic axis of the ICC using a multi-site electrode array. We then plotted the temporal pattern of spiking activity as post-stimulus time histograms (PSTHs) and compared them with the different envelopes of the presented vocalizations. Each PSTH corresponds to a recording site within a given BF region. Each envelope of a vocalization corresponds to the motion (i.e., velocity in our study) of the corresponding BF location along the basilar membrane that is outputted from a dual resonance non-linear (DRNL) model, which was developed for the guinea pig peripheral auditory system by Meddis et al. (2001) and Sumner et al. (2003). The DRNL model estimates the transformation of the sound stimulus through the cochlea after it is passes through a set of middle ear filters, outputting a signal for each basilar membrane location. We define the DRNL output as the envelope of each of these BF-matched signals.

### VOCALIZATION RECORDINGS

**FIGURE 1:**
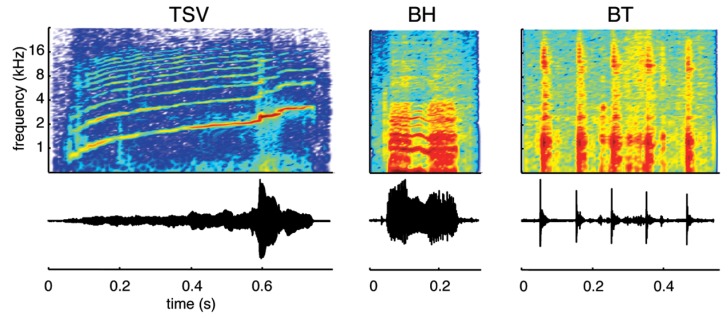
**Vocalization waveforms and spectrograms.** Three different types of vocalizations were used as acoustic stimuli: a temporally and spectrally varying stimulus (TSV), a broadband with harmonic components stimulus (BH), and a broadband and transient stimulus (BT). All three cover a wide spectral range but differ in their spectral and temporal characteristics. The vocalizations presented to the animals were bandpass filtered (500 Hz to 40 kHz) and calibrated with respect to the speaker–ear interface.

We recorded species-specific vocalizations from adult guinea pigs (Dunkin Hartley; Harlan Laboratories, Venray, Netherlands), which were the same breed of animals later used for the neurophysiological recording experiments. The different vocalizations were recorded using a calibrated omnidirectional condenser microphone (model MK202E, Microtech Gefell, Germany), amplified through a TDT MA3 microphone amplifier (Tucker-Davis Technology, Alachua, FL, USA), digitized with a TDT RX6 system (97.5 kHz sampling frequency, 24-bit resolution), and processed using custom Matlab software (MathWorks, Natick, MA, USA). To avoid background noise, the recordings were performed in a sound attenuating recording box (Human Tec, Leopoldshöhe/Greste, Germany). During processing of the recorded vocalizations, we only selected those that exhibited a high signal-to-noise ratio and with minimal interference from movement (determined through video recordings) or vocalizations from other animals. We also ensured that a 50 ms quiet period existed before and after each vocalization for the sound stimuli used in the experiments. A 25 ms cosine ramp was used at the onset and offset of each of these stimuli (i.e., during those 50 ms portions) to avoid any transient steps that could affect the neural responses recorded in the ICC. In addition, the signals were bandpass filtered between 500 Hz and 40 kHz, which corresponded to the effective range of our TDT CF1 speakers. Although there have been up to 11 types of calls documented for the guinea pig (Harper, 1976; Suta et al., 2003; Philibert et al., 2005; Grimsley et al., 2012), we recorded ICC response patterns to only three vocalizations in this study due to time limitations during each experiment (**Figure 1**). We selected these three vocalizations because they exhibit a wide range of spectral and temporal features characteristic of the different types of natural sounds generated by guinea pigs. The first one consists of a harmonic upward sweep with varying frequency components over time, which we call a temporally and spectrally varying stimulus (TSV). The second one contains two broadband components surrounding a shorter harmonic portion in the middle, which we call a broadband with harmonic components stimulus (BH). The third one consists of several short bursts that each cover a broad frequency range, which we call a broadband and transient stimulus (BT).

### SOUND CALIBRATION

Pure tones, broadband noise, and vocalizations were presented through TDT CF1 speakers coupled to the left ear through a hollow ear bar. For calibration, the same setup was used with a 0.25-inch condenser microphone (ACO Pacific, Belmont, CA, USA) where the tip of the ear bar was inserted into a short plastic tube with the microphone inserted into the other end. The tube matched the dimensions of a typical guinea pig ear canal. Pure tones and broadband noise stimuli were calibrated by correcting for the magnitude response obtained from playing pure tones through the speaker–ear system. However, to calibrate the vocalizations, the phase response also had to be corrected. For this purpose, we applied a normalized least mean squares (NLMS) adaptive filter to estimate the complete inverse transfer function of the speaker–ear system (Moonen and Proudler, 1998). We calibrated each vocalization for different levels ranging from 30 to 70 dB SPL (10 dB steps) that corresponded to the peak value from a 20 ms sliding root mean square window of each signal.

### ANESTHESIA AND SURGERY

All experiments were performed on male or female albino guinea pigs (458–749 g; Dunkin Hartley; Harlan Laboratories, Venray, NL). We initially anesthetized the animals with an intramuscular injection of ketamine (40 mg/kg) and xylazine (10 mg/kg) with periodic supplements to maintain a non-reflexive state. Atropine sulfate (0.05 mg/kg) was injected subcutaneously throughout the experiment to reduce bronchial secretion. A warm water heating blanket controlled by a rectal temperature probe was used to keep the body temperature at 38 ± 0.5^°^C. All experiments were carried out in accordance with the German law for animal protection and were approved by the regional government (Landesamtes für Verbraucherschutz und Lebensmittelsicherheit registration number 33.9-42502-04-09/1666).

Before surgery, we recorded auditory brainstem responses (ABRs) using 10 ms pure tones as acoustic stimuli (1, 4, 8, 16, and 32 kHz; 1 ms rise–fall ramp times) and low impedance subcutaneous electrodes positioned at the left mastoid (signal), vertex (reference), and frontal skull region (ground). We excluded any animals with hearing thresholds greater than 30 dB SPL at 8 kHz or abnormally shaped audiograms (typical examples shown in Gourevitch et al., 2009). After fixing the animal into a stereotaxic frame (David Kopf Instruments, Tujunga, CA, USA), we exposed the right occipital lobe. To position a multi-site electrode array (NeuroNexus Technologies, Ann Arbor, MI, USA) into the ICC, we partially aspirated the occipital cortex and exposed the right inferior colliculus surface (Bledsoe et al., 2003; Snyder et al., 2004). The array consisted of two shanks (500 μm separation) each with 16 linearly spaced sites (100 μm spacing, ß400 μm^2^ area). The array was inserted at a 45^°^ angle to the sagittal plane with each shank aligned along the tonotopic axis of the ICC (Malmierca et al., 1995; Snyder et al., 2004; Lim and Anderson, 2006). The ground wire was placed in the neck muscles. After placement of the array, the brain was covered with agarose gel. During the experiment, the array was repositioned two to three times.

All experiments used for this paper had a total duration between 12 and 16 h. As described above, ABRs were obtained at the beginning of each experiment, which required approximately 45 min. The surgery required about 2–3 h. The neural recordings from the ICC had a duration between 8 and 12 h. All animals were euthanized after each experiment by decapitation under deep anesthesia.

### STIMULATION AND RECORDING SETUP

All experiments were performed in an acoustically and electrically shielded chamber and controlled by a computer interfaced with TDT System 3 hardware using custom software written in Matlab. All stimuli were calibrated as described above. To aid in positioning the electrode array, we presented various levels of pure tones and broadband noise that were 50 ms in duration with 5 ms and 0.5 ms rise–fall ramp times, respectively, to elicit acoustic-driven activity in the contralateral ICC. Once the array was correctly positioned within the ICC, each vocalization was presented at different levels ranging from 30 to 70 dB SPL in 10 dB steps and randomized across all stimuli (20 trials for each stimulus). Inter-stimulus intervals were at least 1.5 s long. We also recorded 20 trials of spontaneous neural activity randomly interleaved with the stimulus trials. All neural signals were passed through analog DC-blocking and anti-aliasing filters from 1.6 Hz to 7.5 kHz. The sampling frequency used for acoustic stimulation was 195 kHz and for neural recording was 24 kHz. The sampling rate of the vocalizations was doubled from 97.5 kHz to match the rate of 195 kHz used for the experiments.

### PLACEMENT OF ELECTRODE ARRAY IN ICC

Post-stimulus time histograms and frequency response maps (FRMs) were plotted online to confirm that the electrode arrays were correctly positioned along the tonotopic axis of the ICC. Details on these analysis methods and example plots for similar types of electrode arrays are presented in previous publications (Lim and Anderson, 2006; Neuheiser et al., 2010). Briefly, for the FRMs we presented pure tones with a length of 50 ms with 5 ms rise–fall cosine ramps, levels ranging from 0 to 70 dB SPL in 10 dB steps, and frequencies ranging from 500 Hz to 50 kHz with six steps per octave. All stimuli were randomly interleaved. Neural recordings were obtained 20 ms before the onset of each stimulus and for a duration of 200 ms. We bandpass filtered the neural signals (300–3000 Hz) and detected spikes on each site that exceeded a manually adjusted threshold above the background activity. We binned the spikes into PSTHs (1 ms bins). The number of trials for broadband stimulation varied whereas four trials were presented for each pure tone and level stimulus for the FRMs. To create a FRM for each site, we calculated the driven spike rate (total minus spontaneous spike rate) within a set PSTH window relative to the stimulus onset (5–65 ms) and plotted that value for each frequency-level combination. Sustained PSTHs in response to broadband stimuli as well as a systematic shift in frequency tuning from low to high frequencies for superficial to deeper sites confirmed that our array was positioned within the ICC.

### OFFLINE DATA ANALYSIS

For the analysis, data from 10 animals were used. The figures in this paper include either single cases or combined data from all 10 animals. We compared the multi-unit spiking activity recorded from different frequency regions of the ICC with the corresponding spectral and temporal pattern of the presented vocalizations. In order to obtain data from many different locations across ICC, we used multi-site arrays that generally record multi-unit activity. It was not possible to reliably isolate single-unit spikes from this multi-unit activity. However, since we are interested in assessing temporal patterns that would guide ICC stimulation, which generally activates clusters of neurons surrounding each electrode site, analysis of multi-unit responses was sufficient for this study. These multi-unit spikes were automatically detected at a threshold of 3.5 times the standard deviation of the background noise recorded on a given site after the signals were bandpass filtered between 300 and 3000 Hz. Only negative peaks were detected as spikes. The total spikes across trials minus the mean spike count across spontaneous trials were calculated for 1-ms bins to obtain a stimulus-driven PSTH for each site and stimulus (i.e., a vocalization at a certain level). We reprocessed the FRM spike data offline in a similar way and calculated the BF by taking the centroid frequency value at 10 dB above the visually determined threshold level from the FRMs, as performed in our previous studies (Lim and Anderson, 2006; Neuheiser et al., 2010). We used this BF measure instead of characteristic frequency (i.e., frequency corresponding to the maximum activity at threshold) because it was less susceptible to noise and more consistent with what we visually estimated from the FRMs.

**FIGURE 2:**
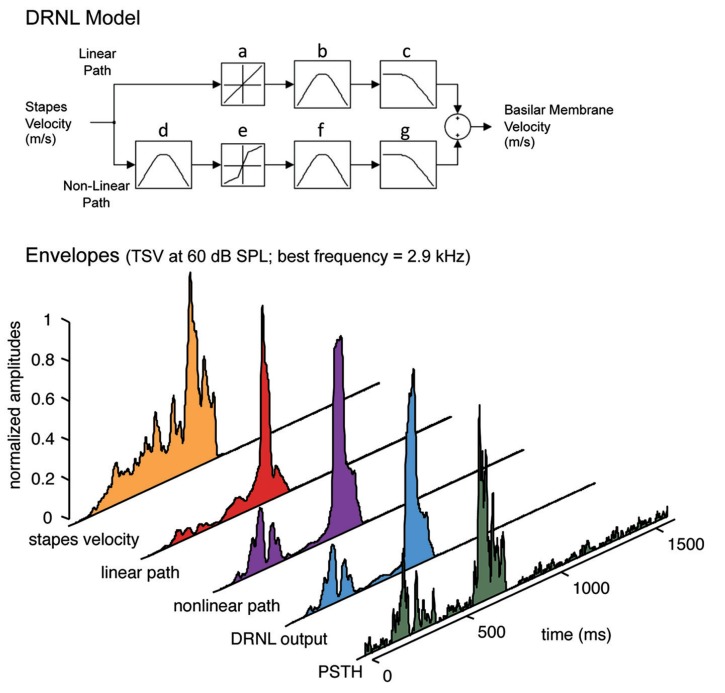
**Peripheral ear modeling and data processing.** *Top*: The dual resonance non-linear (DRNL) model is fit with guinea pig parameters. The model simulates the peripheral processing from the stapes input to the output of a specified best frequency location along the basilar membrane. The model consists of a linear and a non-linear path, each with a set of gammatone bandpass filters (b, d, f) and Butterworth lowpass filters (c, g) that vary depending on the specified best frequency. In addition, the non-linear path of the model contains a broken stick compression function (e; also varying with best frequency and in contrast to the linear function in a) to estimate the non-linear effects of the cochlea. *Bottom*: The signals are extracted for each part of the DRNL model and compared with the neural response for a single ICC site. Before applying the DRNL model, the signal is first inputted through a middle ear filter (two Butterworth bandpass filters from 4 to 25 kHz and from 700 Hz to 30 kHz) and scaled from sound pressure to *stapes velocity*. The output of the DRNL model is obtained from summing the *linear path* and *non-linear path* components. The envelope of the DRNL model (i.e., what we define as the *DRNL output*) is then extracted using the Hilbert transform method and smoothed by a 10 ms sliding window average to obtain the slower fluctuations of the signal (see Materials and Methods for further details). The envelopes for the other signal components were extracted in the same way and displayed in the figure for visualization. The post-stimulus time histogram (PSTH), which was also smoothed with a 10 ms sliding window average, shows the neural activity recorded in a 2.9 kHz frequency lamina in response to TSV shown in **Figure 1**. The best frequency of 2.9 kHz was used for the DRNL model to obtain the different envelope signals shown in the figure, except for stapes velocity, which is independent of best frequency.

The PSTH for each site with a specific BF and for a given vocalization was then compared with its BF-matched DRNL output. The DRNL output corresponds to the envelope of the basilar membrane velocity at a given BF location. The DRNL model of the peripheral auditory system was developed by Meddis et al. (2001) and modified for the guinea pig by Sumner et al. (2003; **Figure 2**). The DRNL model uses stapes velocity as the input. To obtain the stapes velocity, we first passed the signal through a middle ear filter, which consisted of a cascade of two Butterworth bandpass filters. One was a second order filter with a bandpass range from 4 to 25 kHz, while the other was a third order filter with a bandpass range from 700 Hz to 30 kHz. Both had unity gain. This middle ear filtering was necessary to reproduce the appropriate activation thresholds observed at the basilar membrane and auditory nerve fibers in the guinea pig (Evans, 1972; Sumner et al., 2003). The output from the middle ear filter was then scaled by a factor of 1.4 × 10^-^^10^ to convert sound pressure into stapes velocity (in m/s). Further details on this middle ear filtering are provided in Sumner et al. (2003). The DRNL model has both a non-linear and a linear path in parallel. The linear path consists of a linear gain, a cascade of three first order gammatone filters, and a cascade of four second order Butterworth lowpass filters. The non-linear path consists of a cascade of three first order gammatone filters, a non-linear broken-stick transfer function to account for compression effects, another cascade of three first order gammatone filters, and a cascade of four first order Butterworth lowpass filter. The outputs of the linear and non-linear paths are then summed together.

Further details on the different parameters and values used for the DRNL model are presented in Sumner et al. (2003) and a freely available Matlab program was obtained from Drs Ray Meddis and Christian Sumner to perform the DRNL simulation in our study. In brief, several parameters were fixed while other parameters changed as a function of BF. To estimate the basilar membrane velocity at a given location, the Matlab program required only the corresponding frequency (i.e., referred to BF in our study) and the vocalization waveform scaled to the desired sound pressure level. The program then calculated and implemented the corresponding bandwidths, cut-off frequencies, gains, and scaling factors required for each component of the DRNL model. This DRNL model has been able to reproduce various tuning properties and non-linear input–output functions at different locations along the basilar membrane, variations in phase response with frequency and level, impulse responses on the basilar membrane, local distortion products, and other peripheral phenomenon that have been measured across several species (Lopez-Poveda and Meddis, 2001; Meddis et al., 2001; Sumner et al., 2003). Combining the DRNL model with an inner hair cell model was also able to reproduce the BF dependence of frequency–threshold tuning curves, rate-intensity functions, iso-intensity functions, compression effects, and basic temporal firing patterns to speech stimuli as measured in the auditory nerve fibers in guinea pig (Sumner et al., 2003; Holmes et al., 2004). As explained in Section “Introduction” for our study, we used the DRNL model without the inner hair cell model because we were initially interested in treating the transformation from the basilar membrane to the ICC as a single black box without incorporating any “neural” components.

Once the output signal from the DRNL model was calculated, we extracted its envelope using the Hilbert transform method, which has shown to successfully extract perceptually relevant envelope features for speech understanding (Smith et al., 2002; Nie et al., 2006). The envelope was downsampled to a resolution of 1 ms to match that of the PSTH. We then further smoothed both the envelope (to obtain what we define as the “DRNL output”) and PSTH using a 10 ms sliding window average, which keeps envelope fluctuations up to ß100 Hz. All PSTH responses and DRNL outputs were normalized by their maximum value (i.e., normalized to 1) for analysis and improved visualization. **Figure 2** presents the normalized envelopes of the different signal components of the model all smoothed with the 10 ms window average to enable direct comparison with the corresponding PSTH. We selected a 10 ms window because we were interested in envelope fluctuations relevant for speech understanding (at least 50 Hz; Shannon et al., 1995) yet within the range of temporal following capabilities shown for ICC neurons, which are mostly below or around 100 Hz across several species, including guinea pig (Rees and Moller, 1987; Rees and Palmer, 1989; Snyder et al., 2000; Joris et al., 2004). Based on visual inspection of the data, we also observed more noticeable differences between the PSTHs and DRNL outputs once we began to decrease the window below 10 ms.

To directly assess similarity, we calculated the peak of the cross-correlation function between a PSTH response and its corresponding DRNL output. If we observed any peak values at a time lag less than 5 ms or above 20 ms, which is typically outside the range for acoustic activation of the ICC (Syka et al., 2000), we visually checked and manually corrected for any artificial correlation values. Additionally, we excluded any cases in which there was low driven activity below a set threshold that prevented reliable comparison with the DRNL output. This set threshold corresponded to four times the standard deviation of the PSTH activity from the spontaneous trials for each recording site. Only PSTHs that consisted of more than 20 bins above that set threshold during the stimulation period were considered valid cases, which matched what we determined through visual inspection of the data. All the PSTHs analyzed in this study spanned a BF range of 500 Hz to 20 kHz. Unless stated in the text or figures, all analyses and plots correspond to a sound level of 60 dB SPL.

## RESULTS

Data were collected from 10 animals. The total number of recording sites in ICC varied for each vocalization and will be specified when presenting the different results.

### ICC RESPONSES TO VOCALIZATIONS

**FIGURE 3:**
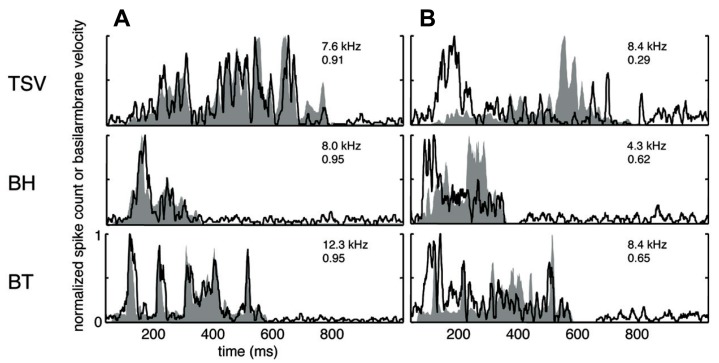
**Correlation examples.** Black lines correspond to the PSTHs and gray shaded areas correspond to the DRNL outputs. The best frequency and the peak correlation value for each recording site are labeled on the top-right of each plot. **(A)** High correlation examples are shown for the three vocalizations presented in **Figure 1**. **(B)** The lowest correlation examples across our data set are shown for the three vocalizations.

A wide range of responses was recorded in the ICC in response to the three different vocalizations. **Figure 3A** presents one example for each vocalization corresponding to one of the highest correlation values. The DRNL output (shaded region) for BT corresponding to a BF of 12.3 kHz is quite similar to the corresponding PSTH response (black line). The PSTH slightly lags behind the DRNL output as expected for the acoustic activation to reach the ICC. The PSTH response was also quite similar to the DRNL output for BH. For TSV, the PSTH response followed almost every peak in the DRNL output, being partially misaligned in magnitude for a few peaks, which is still remarkable considering the large number of peaks and complex pattern of the stimulus envelope compared to those for BT and BH. On the other extreme, we observed PSTH responses that were quite different from the DRNL output in which the temporal patterns and peaks were completely misaligned. **Figure 3B** presents one example for each vocalization with the lowest correlation value across our entire data set. These results suggest that some ICC neurons can closely follow the envelope structure of natural stimuli, while other neurons exhibit different spiking patterns not locked to the stimulus envelope.

**FIGURE 4:**
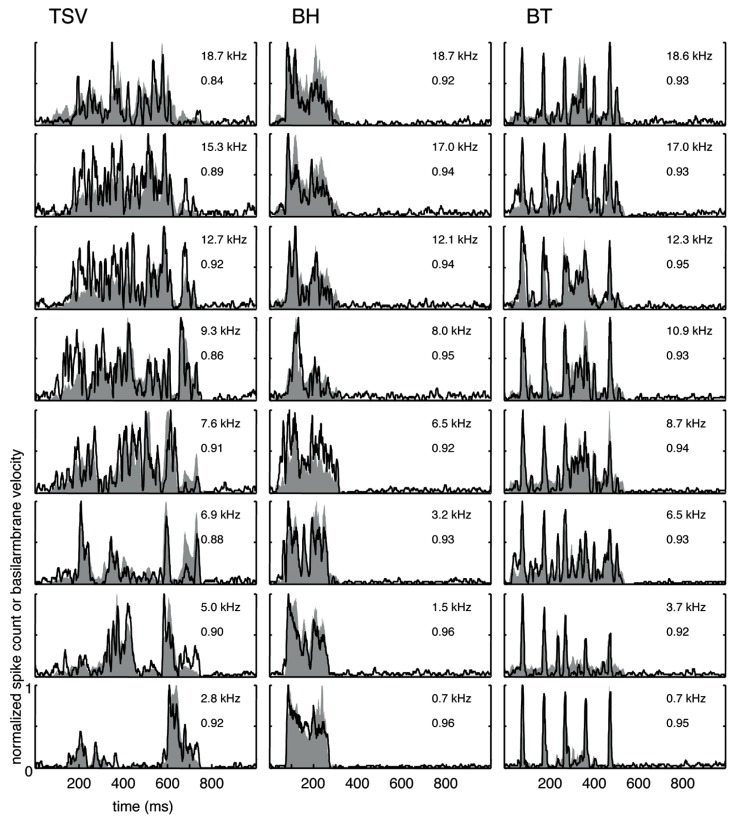
**Maximum correlations.** Examples with the highest correlation values across our data set for different frequency regions are shown for the three vocalizations. Black lines correspond to the PSTHs and gray shaded areas correspond to the DRNL outputs. The best frequency and the peak correlation value for each recording site are labeled on the top-right of each plot.

**FIGURE 5:**
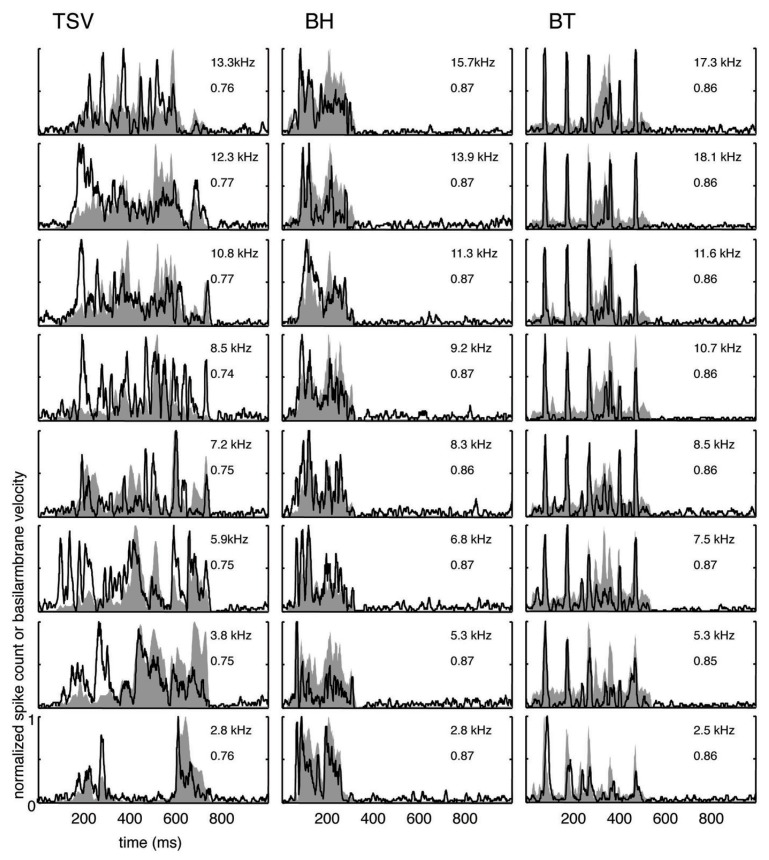
**Median correlations.** Examples with correlation values around the median value across our data set are shown for different frequency regions for the three vocalizations. The median correlation value is 0.75 for TSV, 0.87 for BH, and 0.86 for BT. Black lines correspond to the PSTHs and gray shaded areas correspond to the DRNL outputs. The best frequency and the peak correlation value for each recording site are labeled on the top-right of each plot.

The cross-correlation provides a measure of the similarity between two curves. A value of one indicates a perfect match between two curves. To provide a qualitative interpretation of the different correlation values observed in our study, we present eight typical examples for each vocalization with the maximum (**Figure 4**) and median (**Figure 5**) correlation values we observed for different frequency regions across our data set. As shown for the maximum correlation cases (for those roughly above 0.9), there are ICC neurons that can generally follow the envelope structure of the basilar membrane in response to different vocalizations and across a wide range of BF regions. The greatest similarity was observed for the BT examples. Some cases for BH and TSV exhibited greater variability between the DRNL output and the PSTH response. However, all of the displayed PSTH responses generally followed the temporal timing of most of the different peaks of the DRNL output, with deviations mainly related to the actual amplitudes of those peaks. The DRNL model provides an estimate of the basilar membrane motion and was fitted with a single set of parameters to represent all BF regions. It is possible that these amplitude discrepancies could be in part due to suboptimal modeling of the amplitude non-linearities at the basilar membrane and would require better fitting of the parameters for each BF region separately. Even if the examples shown in **Figure 4** are an underestimation of the actual similarity between the DRNL outputs and PSTH responses, they still demonstrate that there are at least some ICC neurons that can closely or approximately follow the envelope structure of natural stimuli.

The median examples across BF regions are presented in **Figure 5**. The median correlation value was 0.75 (*n* = 239), 0.87 (*n* = 203), and 0.86 (*n* = 277) for TSV, BH, and BT, respectively. Median PSTH examples for BH and BT can approximately follow the DRNL outputs though there are greater differences between the peak amplitudes compared to those in the maximum correlation group. The differences in temporal timing and amplitude of the peaks between the PSTHs and DRNL outputs are greater for TSV than for the other two calls.

Based on all the different ICC examples from our data set, including the typical ones shown in **Figures 4** and **5**, correlation values above 0.85 correspond to ICC responses that approximately or closely follow the DRNL output. In particular, 15% (36 of 239), 60% (122 of 203), and 58% (160 of 277) of the sites for TSV, BH, and BT, respectively, satisfy this criterion. Out of the 36 TSV cases with high correlation values (≥0.85), 64% had high correlation values also for BH and BT, while 78% had high correlation values for at least one of the other calls. Additionally, 75% (92 of 122) of the BH cases with high correlation values also had high correlation values for BT. Therefore, the same ICC neurons can follow the envelope structure for two or all three of the vocalizations used in our study demonstrating that there is a subset of ICC neurons designed to temporally code for a diverse range of spectral and temporal patterns relevant for natural sound processing. These neurons generally follow the envelope structure for BH and BT better than for TSV, which may relate to the more complex and time-varying spectral pattern of TSV.

### SPECTRAL AND TEMPORAL FEATURES

**FIGURE 6:**
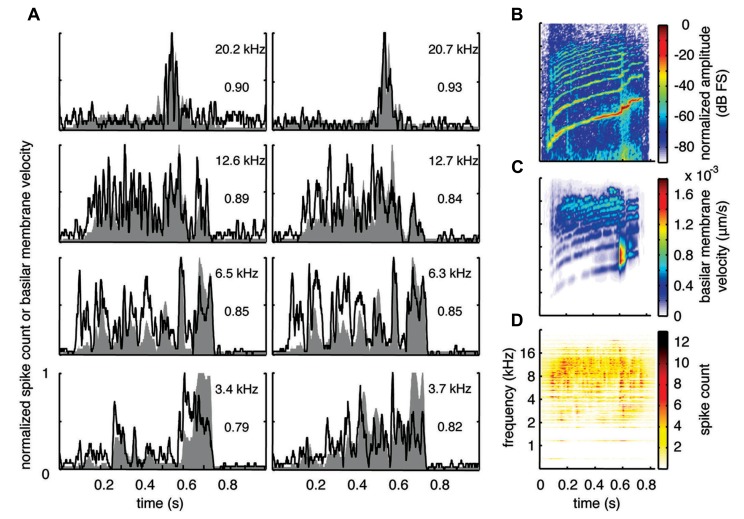
**Spectrotemporal representation forTSV.** Neural activity was recorded across a wide range of best frequency regions from 10 animals and compared to the corresponding frequency-specific DRNL outputs of the stimulus. **(A)** DRNL outputs and PSTHs for four different frequency laminae (shown in rows) corresponding to two different shanks (shown in columns) from the same recording probe. Black lines correspond to the PSTHs and gray shaded areas correspond to the DRNL outputs. The best frequency and the peak correlation value for each recording site are labeled on the top-right of each plot. **(B)** Spectrogram of the stimulus using the short-time Fourier transform. **(C)** Spectrogram made from the frequency-specific envelopes outputted from the DRNL model. Each row of the plot is an envelope outputted from one discrete DRNL frequency channel. Note that the different frequency channels of the DRNL model overlap in frequency ranges and thus smear the spectrogram plot in the frequency dimension. **(D)** Neural representation of the spectrotemporal features of activity recorded across all 239 sites. Each row is a PSTH recorded from each ICC site for a specific best frequency region. dB FS decibels relative to full scale in which 0 dB FS corresponds to the maximum value.

**FIGURE 7:**
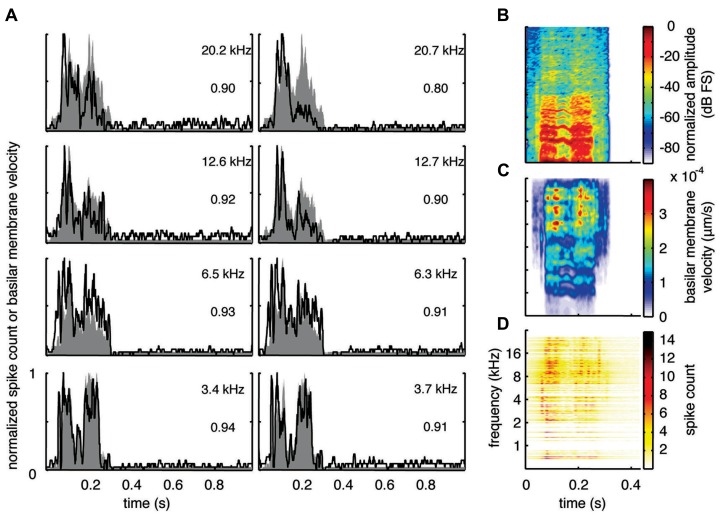
**Spectrotemporal representation for BH.** Neural activity was recorded across a wide range of best frequency regions from 10 animals and compared to the corresponding frequency-specific DRNL outputs of the stimulus. **(A)** DRNL outputs and PSTHs for four different frequency laminae (shown in rows) corresponding to two different shanks (shown in columns) from the same recording probe. Black lines correspond to the PSTHs and gray shaded areas correspond to the DRNL outputs. The best frequency and the peak correlation value for each recording site are labeled on the top-right of each plot. **(B)** Spectrogram of the stimulus using the short-time Fourier transform. **(C)** Spectrogram made from the frequency-specific envelopes outputted from the DRNL model. Each row of the plot is an envelope outputted from one discrete DRNL frequency channel. Note that the different frequency channels of the DRNL model overlap in frequency ranges and thus smear the spectrogram plot in the frequency dimension. **(D)** Neural representation of the spectrotemporal features of activity recorded across all 203 sites. Each row is a PSTH recorded from each ICC site for a specific best frequency region. dB FS decibels relative to full scale in which 0 dB FS corresponds to the maximum value.

With the two-shank array, it was possible to simultaneously record neural activity from two sites within a similar isofrequency lamina of the ICC in addition to multiple sites across different frequency regions. **Figures 6A, 7A,** and **8A** show data recorded from four different frequency laminae from the same sites and animal in response to the three different vocalizations. The DRNL output is approximately similar across the two sites in each lamina (i.e., slightly different due to slightly varying BF values). However, the PSTH responses could exhibit more drastic differences between the two sites even though they had approximately similar BF values. It appears that some peaks in the DRNL output are better represented by one site versus the other site, and vice versa. For example in **Figure 6A** for the 12.6–12.7 k Hz lamina, the site in the right column exhibited a PSTH response that more closely followed the peaks at ß0.6–0.7 s compared to the site in the left column. In addition, as mentioned above, the PSTH response more closely matched the DRNL output for BT and somewhat for BH compared to TSV. For example, this comparison can be observed for the same 6.5 kHz site in **Figures 6A** (TSV), **7A** (BH), and **8A** (BT).

**FIGURE 8:**
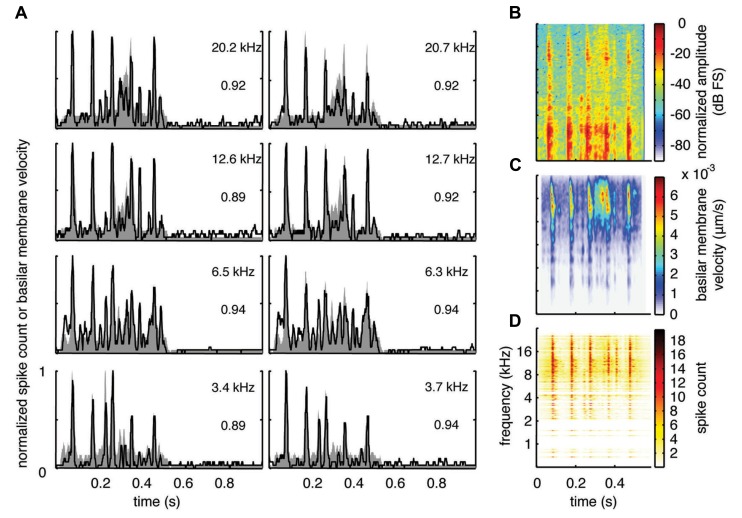
**Spectrotemporal representation for BT.** Neural activity was recorded across a wide range of best frequency regions from 10 animals and compared to the corresponding frequency-specific DRNL outputs of the stimulus. **(A)** DRNL outputs and PSTHs for four different frequency laminae (shown in rows) corresponding to two different shanks (shown in columns) from the same recording probe. Black lines correspond to the PSTHs and gray shaded areas correspond to the DRNL outputs. The best frequency and the peak correlation value for each recording site are labeled on the top-right of each plot. **(B)** Spectrogram of the stimulus using the short-time Fourier transform. **(C)** Spectrogram made from the frequency-specific envelopes outputted from the DRNL model. Each row of the plot is an envelope outputted from one discrete DRNL frequency channel. Note that the different frequency channels of the DRNL model overlap in frequency ranges and thus smear the spectrogram plot in the frequency dimension. **(D)** Neural representation of the spectrotemporal features of activity recorded across all 277 sites. Each row is a PSTH recorded from each ICC site for a specific best frequency region. dB FS decibels relative to full scale in which 0 dB FS corresponds to the maximum value.

For this study, we did not systematically investigate the effects of different locations along a given ICC lamina on temporal following properties. However, we pooled data across all our sites within and across different frequency regions to assess if the population PSTH could resemble the spectrotemporal patterns observed in the spectrogram for each vocalization. **Figure 6B** displays the spectrogram for TSV. In **Figure 6C**, the DRNL outputs across different frequency regions ranging from 500 Hz to 20 kHz were concatenated to create a plot that resembles a spectrogram. By comparing **Figures 6B,C,** it can be seen that the middle ear filtering and DRNL model attenuates the lower frequency components of the stimulus (i.e., relatively enhances the higher frequency components) while slightly smearing the stimulus in the frequency dimension due to the overlapping frequency bands along the basilar membrane. The stimulus has also been smoothed in the time domain by the Hilbert transform method and a 10 ms sliding window average. In **Figure 6D**, we concatenated all the PSTHs recorded across the ICC in response to TSV at 60 dB SPL and obtained the population PSTH. The population PSTH more closely matches the DRNL spectrogram (**Figure 6C**) than the actual stimulus spectrogram (**Figure 6B**), especially for the weaker activity within the lower frequency regions. Similarly, general spectrotemporal features in the DRNL spectrogram can be observed in the population PSTH for both BH (**Figure 7**) and BT (**Figure 8**). Note that we did not have many sites for BFs below 2 kHz and thus cannot interpret how well ICC neurons follow the sound envelope for those lower frequencies. Interestingly, a few low BF sites had strong activity even though there was weak sound energy in the DRNL output. Strong activity in low frequency regions of the ICC associated with low stimulus energy has also been observed in a previous study (Suta et al., 2003), and may reflect the non-linear transformations that have occurred from the periphery up to ICC.

### AMPLITUDE NON-LINEARITIES IN ICC ACTIVITY

**FIGURE 9:**
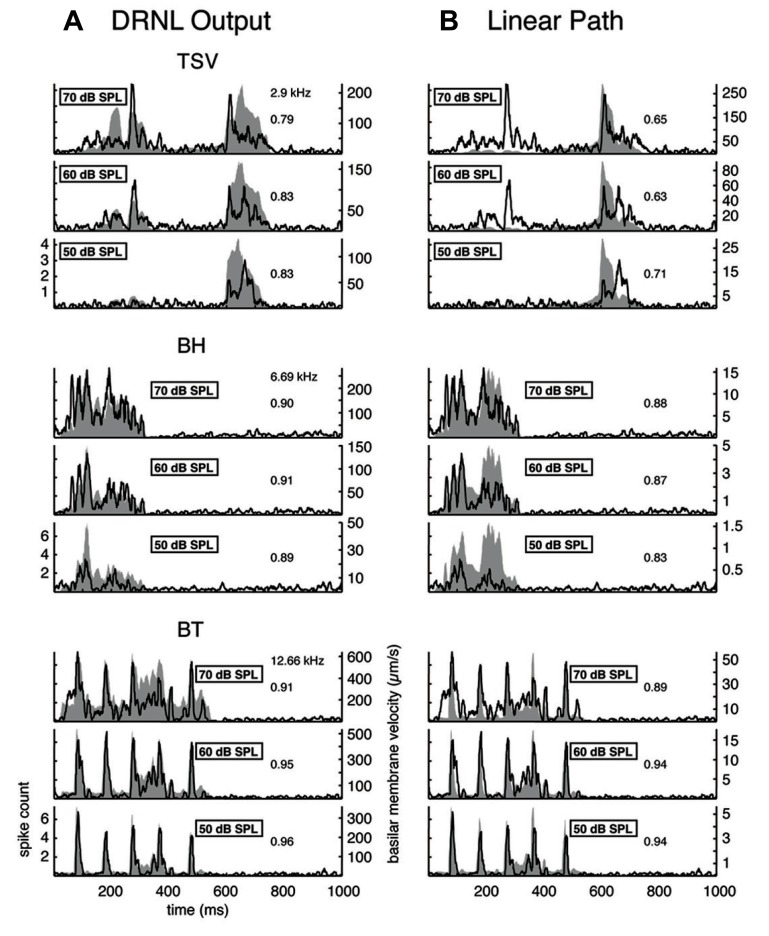
**Comparison of DRNL output versus the linear path output.** The PSTH responses are plotted for three different levels along with the DRNL output **(A)** and the linear path component of the DRNL model **(B)** for all three vocalizations. Black lines correspond to the PSTHs and gray shaded areas correspond to the DRNL outputs. The best frequency and the peak correlation value for the recording sites are labeled on the top-right of the plots.

The DRNL model incorporates amplitude compression through the non-linear path in which smaller amplitude components can increase to a greater extent than larger amplitude components, as has been observed in basilar membrane and auditory nerve activity in animals (Yates et al., 1990; Cooper and Yates, 1994; Meddis et al., 2001; Sumner et al., 2003). To assess the contribution of this compression component, we compared the DRNL output with the linear path output (the red component shown for the envelopes in **Figure 2**). The first column of **Figure 9** presents the DRNL output for the different vocalizations at several levels while the second column displays just the linear path output. Note that the linear path output is identical across levels for a given vocalization except for a scaling factor, which can be seen as the *y*-axis range changes for each level. All amplitude values change proportionally within the linear path. For TSV, as the stimulus level increases from 50 to 70 dB SPL, the smaller PSTH values of the linear path (e.g., those between ß100 and 400 ms) increase to a greater extent than the larger PSTH values. The DNRL model compensates for some of these non-linear effects. However, the changes in amplitude with increasing level do not appear to be fully captured by the DNRL model. Different types of compressive effects are also observed for the BH and BT examples. For BH, the DRNL model produces a pattern that generally matches the PSTH response better than that of the linear path (e.g., the peaks around 200 ms), but not for all of the peaks. For BT, there is also a mixture of effects in which the DRNL model performs better for some peaks while the linear path performs better for other peaks. Overall, the DRNL model is not able to fully produce the envelope patterns observed for all of our recorded ICC sites across different levels. It is important to note, as shown in the next section, that there are still some ICC neurons that can closely follow the DRNL output for different levels. However, we present the complex patterns in **Figure 9** to show that there are neural features that are not being fully predicted by the DRNL model.

The DNRL model has provided good estimates for the compression effects observed for the basilar membrane and auditory nerve in guinea pig (Meddis et al., 2001; Sumner et al., 2003). We also observed high correlation values between the PSTHs and DRNL outputs for multiple ICC sites **Figures 3A** and **4**) indicating that some ICC neurons are following the motion at the basilar membrane predicted by the DNRL model. Therefore, the complex changes in response amplitude with increasing level in ICC neurons that are not predicted by the DRNL model could be associated with actual neural transformations that have occurred from the periphery up to the ICC.

### SUMMARY OF CORRELATION VALUES

**FIGURE 10:**
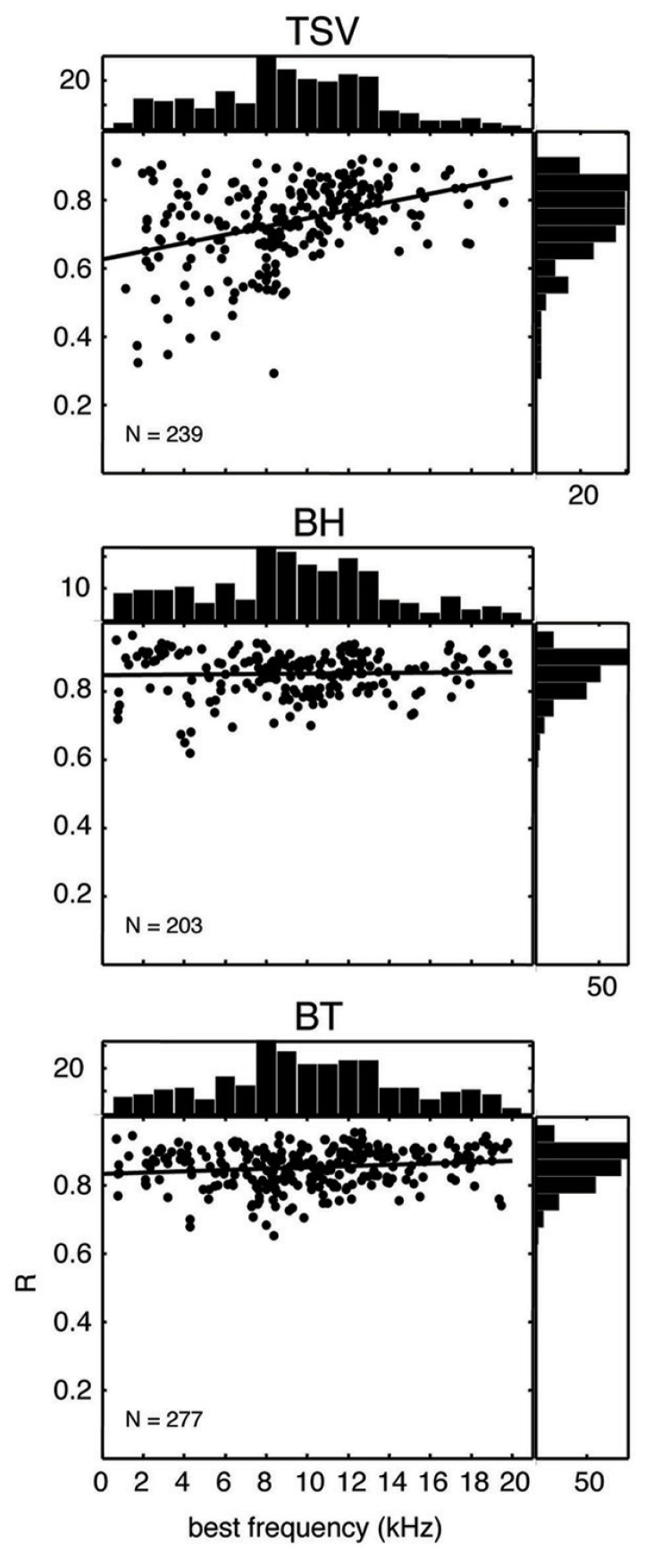
**Scatter plots for correlation values versus best frequency.** The peak correlation values (*R*) is plotted for ICC sites with different best frequencies for all three vocalizations from 10 animals. The histogram on top of each plot shows the distribution of best frequencies across all the ICC sites, while the histogram on the right shows the distribution of correlation values. The solid line in each plot corresponds to the least-squares fit to the data. *N* denotes number of total ICC sites for each vocalization.

In **Figure 10**, the correlation values for all our recording sites are plotted for each vocalization as a function of BF. The correlation values for BH and BT show consistently high values over the entire range of BFs and with values typically above 0.7. In contrast, there was a much wider range of correlation values for TSV, especially for lower BF sites. There appears to be a greater number of low BF neurons that are not capable of following the envelope for TSV even though many of those same neurons could reliably follow the envelopes for BH or BT.

**FIGURE 11:**
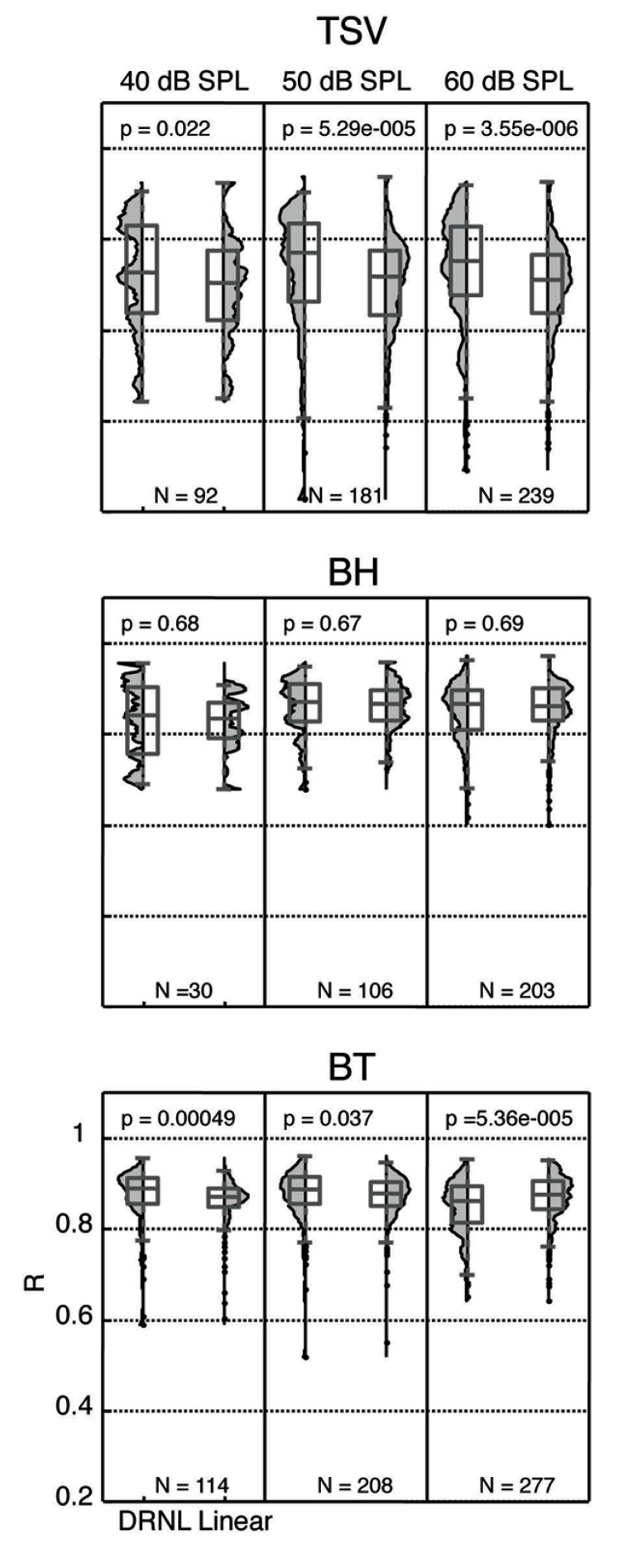
**Distribution of correlation values for different levels.** The peak correlation values (*R*) are plotted for both the linear path and DRNL outputs for three different stimulus levels for each vocalization from 10 animals. The *R* values were significantly different between the linear path output and the DRNL output for some cases (*p* < 0.05, Wilcoxon rank-sum test). *N* denotes number of total ICC sites for each condition.

We re-plotted the correlation values from **Figure 10** in a histogram format independent of BF to enable comparison with different stimulus levels with and without the non-linear path (**Figure 11**). For TSV, the DRNL output exhibited significantly higher correlation values compared to those of just the linear path at all three levels in which there was greater significance for higher levels. The BT also exhibited significantly higher correlation values for the DRNL output compared to those of the linear path but only at 40 and 50 dB SPL. In contrast to TSV, the significance values for BT actually decreased with increasing level such that the correlation values were significantly lower for the DRNL output versus the linear path output at 60 dB SPL. None of the levels for BH exhibited any significant differences in correlation values between the DRNL output and the linear path output. It is unclear as to why adding the non-linear component of the DRNL model improved the similarity between the DRNL outputs and the PSTH responses for TSV and BT but not for BH. TSV exhibits time-varying temporal and spectral patterns while BT exhibits more transient and broadband patterns. Considering that BH consists of a combination of patterns found in both TSV and BT, we also expected significantly higher correlation values for the DRNL output versus the linear path output for BH, at least for some levels.

In the previous section, we showed that ICC neurons could exhibit complex neural patterns across different levels that were not fully produced by the DRNL model. As shown in **Figure 11**, there are still ICC sites that exhibit high correlation values across different levels (i.e., for 40, 50, and 60 dB SPL). Due to an insufficient number of data points, we did not include analysis for 30 and 70 dB SPL in **Figure 11**. Interestingly, there was a moderate percentage of ICC sites that exhibited high correlation values (≥0.85) across all three levels for BH (38.5%; 10 out of 26) and BT (56.6%; 64 out of 113). For TSV, only 4.5% (4 out of 88) of the sites exhibited high correlation values across all three levels. It is important to note that each ICC site may not have data for all three levels, and thus the total number of sites for each vocalization listed in parentheses above is smaller than the total number (i.e., for *N*) listed in **Figure 11** for each level. Overall, these data suggest that there is at least a small subset of ICC neurons that are capable of coding for the envelope of the vocalizations across different levels as predicted by the DRNL model.

## DISCUSSION

The three vocalizations used in this study were specifically selected to ensure that the neurons were activated with a broad spectrum of temporal and spectral patterns that the animals typically encounter on a daily basis. Based on our results, ICC neurons exhibit a wide range of neural patterns in response to these species-specific vocalizations. Previous studies have also shown that ICC neurons exhibit a wide range of neural patterns in response to artificial and natural stimuli (Rees et al., 1997; Krishna and Semple, 2000; Suta et al., 2003; Zheng and Escabi, 2008; Holmstrom et al., 2010) suggesting that different and complex transformations have occurred from the auditory nerve through the central auditory system. Cochlear neurons can reliably follow the temporal pattern of an acoustic stimulus, even up to several thousand hertz (Johnson, 1980; Joris et al., 2004). In contrast, ICC neurons generally follow the temporal patterns up to only a few hundred hertz (Rees and Moller, 1987; Krishna and Semple, 2000; Frisina, 2001; Langner et al., 2002). It has been proposed that the fast features of the stimulus have been converted into a complex rate code and/or population spiking pattern across neurons (Langner and Schreiner, 1988; Joris et al., 2004; Wang et al., 2008; Huetz et al., 2011). For the slower temporal components representing the envelope structure of the stimuli, there also appears to be a rate and/or place code in which different neurons are most sensitive to different amplitude modulation rates (Langner and Schreiner, 1988; Langner et al., 2002). Therefore, it is evident that the ICC is not simply replicating what occurs at the auditory nerve level, especially after passing through several synapses along the ascending auditory pathway. However, these transformations do not mean that there are no ICC neurons that can still reliably or approximately follow the envelope structure of natural stimuli. In this study, we present several cases in which ICC neurons closely followed the frequency-specific envelope of different vocalizations, at least down to a smoothing window of 10 ms (up to ß100 Hz). The following capabilities were greater for BT and BH compared to TSV. Interestingly, a large percentage of ICC neurons that followed the TSV stimulus with high correlation values were also able to closely follow the envelope structure of the other two calls. Based on these findings and previous studies, we propose that there are parallel pathways through the ICC and up to the auditory cortex in which some ICC neurons exhibit complex and transformed spiking patterns while other neurons still transmit a robust temporal representation matching the envelope of a diverse range of complex stimuli. The functional interpretation and supporting evidence for this view is further described below after discussing a few limitations of the study. We also discuss the implications of these findings for AMI implementation.

### METHODOLOGICAL LIMITATIONS

There are a few limitations associated with the methods of our study that need to be discussed when interpreting the results. These limitations are associated with the use of a peripheral ear model to estimate the frequency-specific envelope at the basilar membrane, the effects of anesthesia, and the use of multi-unit activity versus single-unit responses.

To obtain the envelope of the basilar membrane at a specific BF location, we estimated the effects of the peripheral ear with a set of middle ear filters, a DRNL model, and an envelope extractor based on the Hilbert transform and a 10 ms smoothing function. The middle ear and DNRL components have been specifically designed for the guinea pig and have been able to estimate many features observed in physiological data: frequency tuning, non-linear input–output functions, variations in phase response with frequency and level, and impulse responses at different BF locations along the basilar membrane; frequency–threshold tuning curves, rate-intensity functions, iso-intensity functions, compression effects for different BF auditory nerve responses; temporal spiking patterns of different BF auditory nerve fibers in response to single and double vowels; and other complex peripheral effects (e.g., two-tone suppression and local distortion products; Meddis et al., 2001; Sumner et al., 2003; Holmes et al., 2004). The Hilbert transform has also been shown to be a successful method for extracting out perceptually relevant envelope features for speech understanding (Smith et al., 2002; Nie et al., 2006). However, we cannot claim that these simplified manipulations will exactly replicate the stimulus envelope at each location along the basilar membrane for all stimuli and across different guinea pigs. Additionally, we had to estimate the BF of each ICC site to then calculate the different BF-dependent parameters for the DRNL model. Our method of BF calculation may not always estimate the true BF for each ICC site. Therefore, these potential biases could have contributed to the wide range of patterns observed in this study, and thus more ICC cases may have actually been capable of following the envelope of the vocalizations than we presented in our results.

Although anesthesia has shown to cause changes in spontaneous activity as well as acoustic-driven temporal and spectral patterns in the auditory cortex, there seem to be minimal changes that occur within the IC, at least when using ketamine (Zurita et al., 1994; Astl et al., 1996; Gaese and Ostwald, 2001; Suta et al., 2003; Syka et al., 2005; Ter-Mikaelian et al., 2007). One study used a combination of ketamine and sodium pentobarbital in gerbils and reported no or minimal changes compared to the awake state for spontaneous activity. In response to artificial stimuli (i.e., pure tones, noise, amplitude modulated sinusoids), they also observed no or minimal changes for first spike latency, variation in first spike latency, trial-by-trial reliability, temporal synchronization capabilities, and acoustic-driven firing rate. Similar to our study, several studies used a combination of ketamine and xylazine in guinea pigs and there appeared to be no or minimal changes compared to the awake state for spontaneous activity, firing patterns in response to pure tones, and tuning properties based on *Q*_10_ values (Astl et al., 1996; Torterolo et al., 2002; Suta et al., 2003). Although a few studies have shown some changes in spontaneous activity and acoustic-driven response patterns (e.g., spike rate, thresholds, latencies) in the IC due to anesthesia (Kuwada et al., 1989; Astl et al., 1996; Torterolo et al., 2002; Tollin et al., 2004), those effects were caused by barbituates that may have altered spiking properties more dramatically than ketamine. Therefore, the use of ketamine in our study is not expected to have dramatically altered the temporal following capabilities of ICC neurons in response to vocalizations. Even if there were some anesthetic effects on our ICC responses, it would seem that the following capabilities would become worse with anesthesia, and thus more ICC neurons may have exhibited high correlation values if our study was performed in awake animals. It seems unlikely that ketamine would somehow improve the following capabilities of ICC neurons to natural stimuli that was not originally possible in the awake state.

We analyzed multi-unit activity instead of single-unit responses. Previous studies have shown that individual ICC neurons have slow membrane time constants (e.g., 12–33 ms) and do not typically fire more than once within a 10 ms window (Sivaramakrishnan and Oliver, 2006; Chen et al., 2012). It is possible that the PSTH response to individual ICC neurons may not be able to fully follow the BF-matched envelope of different vocalizations because they cannot continuously keep up with the entire pattern over time. However, multi-unit activity consisting of clusters of neurons surrounding a given ICC site may exhibit greater following capabilities because each neuron could fill in different parts of the stimulus envelope. As a result, the correlation values and examples presented in our study may be higher than what are typically observed for individual neurons. Regardless, some individual ICC neurons must still be able to follow the stimulus envelope, even if just certain portions of the envelope, so that together with other neurons they can elicit the types of patterns observed in our study. It will be interesting to investigate this possibility and if different neurons located along the same ICC lamina are designed to accurately represent the envelope structure of natural stimuli as a temporal code across a cluster or population of neurons. At least for AMI implementation, electrical stimulation of a given site will activate a cluster of neurons surrounding that site. Therefore, AMI stimulation may activate enough neurons surrounding each site to still achieve the envelope following capabilities that were observed for a subset of our multi-unit recordings.

### PARALLEL SUB-LEMNISCAL CODING PATHWAYS

Anatomical studies have shown that spatially distinct functional zones exist within the ICC that receive different combinations of inputs from lower brainstem centers (Roth et al., 1978; Brunso-Bechtold et al., 1981; Shneiderman and Henkel, 1987; Oliver et al., 1997; Cant and Benson, 2003, 2006; Loftus et al., 2004). Furthermore, electrophysiological studies have demonstrated that different regions along the isofrequency ICC laminae process sound information (e.g., threshold, frequency tuning, frequency sweep speed, best modulation frequency, latency, binaurality) in different ways (Semple and Aitkin, 1979; Stiebler, 1986; Langner and Schreiner, 1987; Schreiner and Langner, 1988; Ehret, 1997; Hage and Ehret, 2003). Therefore, it is expected that different ICC neurons would exhibit varying spiking patterns to the same sound stimulus.

What is particularly interesting about our results is that some ICC neurons could closely follow the envelope of the vocalizations while other neurons exhibited complex and dissimilar spiking patterns. Based on the studies described above, it is not surprising that we observed these differences in spiking patterns across different ICC neurons. The question arises as to whether this subset of neurons that can closely follow the envelope of natural stimuli are scattered throughout the ICC or if these neurons are located in a specific region within the ICC. The latter scenario would be advantageous for the AMI in which the electrode array could be implanted within a specific ICC region to potentially transmit sufficient envelope cues to higher perceptual centers. In our study, we did not histologically confirm the location of our recording sites throughout the ICC to answer that question. However, there are several anatomical and physiological studies described below that have revealed a specific ICC region that exhibits more robust temporal (and spectral) coding properties than the other ICC regions, and thus may correspond to the location of the neurons that closely followed the envelope of the vocalizations in our study.

In gerbils, it was shown that neurons from the cochlear nuclei and nuclei of the lateral lemniscus project throughout the ICC, while inputs from the superior olivary complex project predominantly to more rostral and lateral locations of the ICC (Cant and Benson, 2006). These anatomical results suggested that at least two distinct regions exist within the ICC: a caudal–medial region and a more rostral–lateral region. The authors further showed that these same regions project to distinct locations along the caudal–rostral (isofrequency) dimension of the ventral division of the medial geniculate nucleus (MGv): the caudal–medial ICC region projects to the caudal third of the MGv while the rostral–lateral ICC region projects to the rostral two-thirds of the MGv (Cant and Benson, 2007). Based on anatomical studies in cats and rats (Rodrigues-Dagaeff et al., 1989; Polley et al., 2007; Storace et al., 2010), the rostral MGv then projects throughout the auditory cortex, including primary auditory cortex (A1), while the caudal MGv projects predominantly to regions outside of A1. Consistent with the spatial organization shown in the anatomical studies and based on physiological studies in cats and guinea pigs, the pathway through more rostral–lateral ICC regions (versus caudal–medial regions along an isofrequency lamina) and more rostral MGv regions (versus caudal regions along an isofrequency lamina) up to A1 exhibits neural activity with lower thresholds, more excitatory activity, greater spatial synchrony across neurons, shorter latencies, and less temporal jitter of spiking to a given stimulus (Rodrigues-Dagaeff et al., 1989; Lim and Anderson, 2007; Straka et al., 2013). This pathway through more rostral MGv regions has also shown to exhibit more precise tonotopy, sharper frequency tuning, and more time-locked spiking to repetitive clicks (Rodrigues-Dagaeff et al., 1989). Together, these findings across different species suggest that there are neurons located in a specific ICC region (i.e., a rostral–lateral location along the isofrequency laminae) that project to rostral MGv up to A1 and are designed to robustly code for sound stimuli. Therefore, the ICC responses that most accurately matched the envelope of the different vocalizations in our study may correspond to neurons within this “rostral–lateral” pathway.

### IMPLICATIONS FOR AMI IMPLEMENTATION

Speech understanding in quiet environments, which is the initial goal for the AMI, is possible with envelope modulations as low as 50 Hz (Shannon et al., 1995; Nie et al., 2006; Xu and Pfingst, 2008). Assuming that the rostral–lateral pathway described in the previous section exists, especially within the human ICC, CI-based strategies may still be effective for the AMI. In particular, if it is possible to target the subset of ICC neurons that can robustly code for the stimulus envelope (up to ß100 Hz), then it may be possible to restore speech perception by stimulating each frequency lamina of the ICC with amplitude-modulated pulse trains matching the corresponding bandpass-filtered envelope of the stimulus based on a DRNL model fitted for humans (Lopez-Poveda and Meddis, 2001).

There are several possibilities as to why the AMI has not yet been able to restore sufficient speech understanding even though a CI-based strategy is being used in the implanted patients. Stimulation of just a single site within each lamina may not activate enough neurons to transmit sufficient envelope cues to higher centers. An alternative possibility is that the current patients were not implanted in the correct region or there is a lack of a specific region to access the subset of ICC neurons identified in our study that could temporally code for the stimulus envelope. Based on the studies described in the previous section, there appears to be a rostral–lateral region along the ICC laminae that could correspond to the location of this subset of robustly coding ICC neurons. However, we need to perform additional studies with histological reconstructions to confirm that the neurons with the highest correlation values across different vocalizations truly correspond to that rostral–lateral region. Furthermore, we observed complex neural patterns across different levels for many ICC neurons. Although we observed some neurons that exhibited high correlation values across multiple levels, we need to investigate if the DNRL model can be expanded to better predict the level-dependent effects across a greater number of ICC neurons since good speech perception may not be achieved with activation of just a few neurons.

More recently, Calixto et al. (2012) has shown that electrical stimulation of a single site in a given isofrequency lamina artificially induces strong inhibitory and suppressive effects within the auditory cortex. However, co-activation of at least two regions within a lamina is able to overcome much of these inhibitory and suppressive properties. The AMI may still be able to use a CI-based strategy to activate the ICC neurons that are capable of following the stimulus envelope, but by stimulating at least two sites within each lamina. Implanting a two-shank electrode array into the ICC to position two sites within each lamina is surgically feasible. Currently, we are investigating and developing a two-shank array device to safely implant and implement in future AMI patients.

## Conflict of Interest Statement

The authors declare that the research was conducted in the absence of any commercial or financial relationships that could be construed as a potential conflict of interest.
